# Dosimetry and verification of ^60^Co total body irradiation with human phantom and semiconductor diodes

**DOI:** 10.4103/0971-6203.37482

**Published:** 2007

**Authors:** Mahmoud Allahverdi, Ghazale Geraily, Mahbod Esfehani, Aliakbar Sharafi, Peyman Haddad, Alireza Shirazi

**Affiliations:** Medical Physics Department of TUMS, Tehran, Iran; * Tehran Medical Science University, Tehran, Iran; ** Radiotherapeutic Oncology Department of Cancer Institute, Tehran, Iran; *** Radiology Department of Iran University, Tehran, Iran; **** Tehran University, Cancer Center of Imam Khomeini hospital, Tehran, Iran

**Keywords:** Dosimetry, human phantom, total body irradiation

## Abstract

Total Body Irradiation (TBI) is a form of radiotherapy used for patients prior to bone marrow or stem cell transplant to destroy any undetectable cancer cells. The dosimetry characteristics of a ^60^Co unit for TBI were studied and a simple method for the calculation of the prescribed dose for TBI is presented. Dose homogeneity was verified in a human phantom. Dose measurements were made in water phantom (30 × 30 × 30 cm^3^), using farmer ionization chamber (0.6 cc, TM30010, PTW) and a parallel plate ionization chamber (TM23343, PTW). Point dose measurements for AP/PA irradiation were measured in a human phantom using silicon diodes (T60010L, PTW). The lung dose was measured with an ionization chamber (0.3 cc, TM31013). The validity of the proposed algorithm was checked at TBI distance using the human phantom. The accuracy of the proposed algorithm was within 3.5%. The dose delivered to the mid-lobe of the lung was 14.14 Gy and it has been reduced to 8.16 Gy by applying the proper shield. Dose homogeneity was within ±7% for all measured points. The results indicate that a good agreement between the total prescribed and calculated midplane doses can be achieved using this method. Therefore, it could be possible to use calculated data for TBI treatments.

Total body irradiation (TBI) is a part of a complex treatment program for aplastic anemia, leukemia, lymphoma and certain other cancers that require chemotherapy and bone marrow transplantation. TBI also helps to cause immunosuppression that is necessary to keep the recipient's immune system from rejecting the bone marrow transplant.[1,2] Hematologists, immunologists, radiotherapists and medical physicists have been trying to increase the success rate of TBI treatments. Improvement requires an understanding of all the clinical, biological and physical aspects.[3] There is no standard TBI technique as hospital radiotherapy departments adopt their own treatment plans that are influenced by the type of treatment facility available.[4] Therefore, different treatment distances, patient set-ups, radiation beams and radiation fields are used in different radiotherapy centers for TBI. Due to the variability of technique, it is not possible to use published data as a reference for another institute. Therefore, if a center wants to implement TBI, it has to measure the basic dosimetric parameters.[3,5]

In TBI set-ups, the patient represents a very irregular and extended field. When such treatment is used as part of the preparation for a bone marrow transplant, it is important to know the dose delivered throughout the body.[6,7] The determination of the dose delivered to the body during TBI is not easy, as direct measurements are impossible.[3] In this study, we present a simple method for absorbed dose determination in ^60^Co TBI using entrance and exit dose readings generated by the semiconductor detectors. The calibration of diodes is also presented here based on a previously described study.[8] The estimated accuracy in dose delivery in TBI as well as standard radiotherapy should be better than ±5%.[3]

Avoiding dose inhomogeneity is very important for TBI because it can lead to failure of TBI through either insufficient dose being delivered to the marrow stem cells or an excessive dose to the critical organs. Therefore, most techniques aim to achieve dose uniformity throughout the whole body while minimizing dose outside the volume especially to critical structures such as the lung.[9,10]

The purpose of this study were (i) to measure some basic dosimetric parameters in the TBI condition in order to check the accuracy in dose delivery. (ii) to develop an algorithm for dose calculation in TBI techniques, and (iii) to investigate dose uniformity in TBI treatment using a human phantom and semiconductor diodes.

## Materials and Methods

The TBI technique was based on a set of parallel-opposed, anterior-posterior fields using a ^60^Co unit with the gantry rotated to project a horizontal beam. The beam collimator was set at 45° so that the field diagonal was projected onto the horizontal plane. The collimator was opened to its maximum field size of 35 × 35 at 80 cm from the source and the Source-Surface Distance (SSD) was 250 cm. The prescribed dose to the umbilicus was 13.5 Gy given in six fractions of 2.25 Gy each twice a day over three days.

### Basic dosimetric parameters (PDD, SC, SP, DR)

In order to calculate the required time for TBI treatment, the following equation was used:

(1) Time=(prescribed dose)/(Sc×Sp×PDD×DR)

where the collimator scatter factor (SC) is defined as the ratio of the output in air for a given field to that for a reference field (10 × 10); the phantom scatter factor (SP) is defined as the ratio of the dose rate for a given field at a reference depth (depth of maximum dose) to the dose rate at the same depth for the reference field size (10 × 10) with the same collimator opening; percentage depth dose (PDD) is expressed as a percentage of absorbed dose at any depth to the absorbed dose at a fixed reference depth (depth of maximum dose) along the central axis of the beam and DR is defined as the output in the phantom for the reference field size (10 × 10) at the depth of the maximum dose.[1,5] SC, SP, PDD and dose rate (DR) were measured in this study.

### Depth dose measurements

Depth dose measurements along the central beam axis were measured in a 30 × 30 × 30 cm^3^ water phantom at a 250-cm source-surface distance using a 0.6-cc Farmer chamber (TM30010, PTW Freiburg) and an electrometer (PTW-UNIDOS). The depth dose fall-off from 2.5 to 25 cm was measured. For surface dose measurements and for relative measurements in the build-up region, a parallel plate chamber (TM23343-PTW Freiburg) was used as previously described.[4] This chamber was put on the water phantom and variable thicknesses of a water-equivalent sheet (T40006, RW3 slab phantom-PTW Freiburg) were placed over the chamber before measurements were taken and corrected for the slight SSD variation.[11,12] A comparison of this depth dose data was made with the measured depth dose at 80 cm SSD and transformed using the Mayneord formula to the TBI distance (SSD = 250 cm).[1,4,11,12]

### Scatter factors

The SC measurement for TBI (with settings: SSD = 250 cm, field size = 35 × 35, collimator angle = 45) was performed using the Farmer ion chamber (0.6 cc) with a cylindrical cap having a radius equal to the electron build-up depth for ^60^Co.[1,5] In order to measure the phantom scatter factor the following equation was used:

(2) $$\text{Sp}=\frac{S_{c,p}}{S_{c}}$$

where in this equation, SC,P is the total scatter factor [defined as the dose rate at a reference depth for a given field size divided by the dose rate at the same point and depth to the reference field size (10 × 10)]. SC,P was acquired from measurements made in the build-up region. The irradiation was based on the calculated time through measurement data and prescribed dose to the umbilicus. In order to check the accuracy in dose delivery, a human phantom which had three sections (head and neck, trunk, hip) was used.[13] A 0.3-cc ion chamber (TM31013, PTW-Freiburg) was imbedded in the phantom at the prescribed point (umbilicus) and the delivered dose was measured.

### Effect of phantom (patient) length

The dose absorbed at any given point depends on the scattering volume surrounding the point.[4,12] Measurements were made to determine the effect of patient length on absorbed dose. The central axis of the water phantom was monitored at several depths while the longitudinal extent of the phantom was changed. Initial measurements started with a 30 × 30 × 30 cm^3^ water phantom and additional material of slab phantoms was added to one side.

### Midplane dose determination Calibration technique

For obtaining the entrance and exit measurements of the human phantom, four p-type diodes (T60010L) were used as dosimeters connected to a MULTIDOS electrometer (T10004). For entrance measurements, calibration diodes were taped on the 30 × 30 × 30 cm^3^ water phantom at an SSD of 250 cm with a 35 × 35 field size. The calibration of these diodes was performed against the 0.6 cc Farmer chamber at depth of 0.5 cm connected to a UNIDOS electrometer (T10001).

The calibration factor (F) was then determined as the ratio of the absorbed dose measured with the Farmer chamber (D) to the reading of the diode (M) in TBI experimental conditions. The exit calibration was the same as the entrance calibration with the exception that the water phantom was turned at 180°.

(3) $$\text{F}=\frac{D}{M}$$

### Target dose calculation

Using combined entrance and exit dose measurements (D_en_, D_ex_), one can estimate the dose delivered to a point placed in the target volume (midplane at umbilicus). In our center, the midplane dose was estimated by three different algorithms: The first algorithm is the arithmetical mean of the entrance and the exit doses. The second algorithm is the geometric means of the entrance and the exit doses.[3] The conversion of entrance and exit doses to any other point is performed using the percentage depth dose corresponding to that point. For example, to obtain D_mid_ from the entrance dose (D_en_), we can multiply D_en_ by PDD_mid_ and to obtain D_mid_ from the exit dose (D_ex_), we can multiply D_ex_ by the ratio of PDD_mid_/PDD_ex_ as shown below:

(4) $$\frac{{\text{PDD}}_{\text{mid}}}{{\text{PDD}}_{\text{ex}}}=\frac{\frac{{\text{D}}_{\text{mid}}}{{\text{D}}_{\text{en}}}}{\frac{{\text{D}}_{\text{ex}}}{{\text{D}}_{\text{en}}}}=\frac{{\text{D}}_{\text{mid}}}{{\text{D}}_{\text{ex}}}$$

(5) $${\text{D}}_{\text{ex}}\text{=}\frac{{\text{PDD}}_{\text{mid}}}{{\text{PDD}}_{\text{ex}}}{\text{=D}}_{\text{mid}}$$

(6) $${\text{D}}_{\text{en}}\times {\text{PDD}}_{\text{mid}}={\text{D}}_{\text{en}}\times \frac{{\text{D}}_{\text{mid}}}{{\text{D}}_{\text{en}}}={\text{D}}_{\text{mid}}$$

To reduce the statistical error in the measurements, the average of Eqs. (5) and (6) was calculated and the third algorithm was expressed as:

(7) $${\text{D}}_{\text{mid}}=\frac{{\text{D}}_{\text{en}}\times {\text{PDD}}_{\text{mid}}+{\text{D}}_{\text{ex}}\times \frac{{\text{PDD}}_{\text{mid}}}{{\text{PDD}}_{\text{ex}}}}{2}$$

where PDD corresponds to the percentage depth dose measured in TBI experimental conditions and D_en_ and D_ex_ are entrance and exit doses respectively. The validity of these algorithms was investigated using the human phantom.[13] Surface doses were measured by diodes and the positioning of these detectors was carefully carried out in order to avoid a shadowing effect. Midplane doses were measured with the 0.3 cc ionization chamber (TM31013).

### Dose uniformity

**Figure 1:**
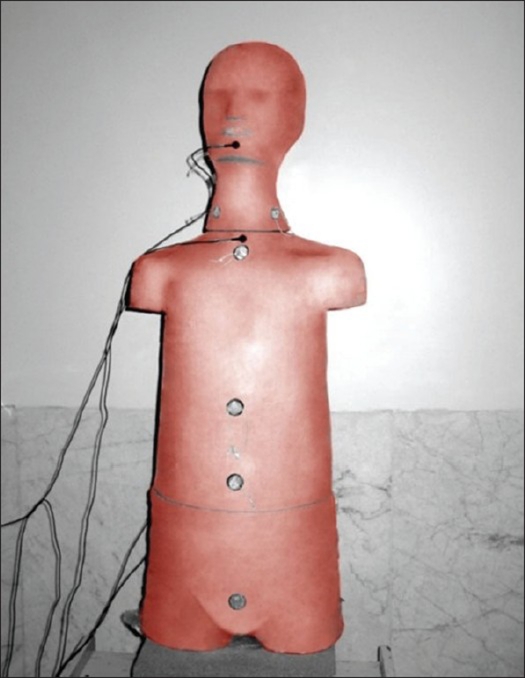
Anterior feature of the human phantom with the diodes

The human phantom was used to investigate dose uniformity throughout the whole body. Diodes were taped on the anterior and posterior surfaces of the human phantom (at six points) and AP/PA radiation was given [Figure 1]. Measured doses at different sites were compared to that at the umbilicus.[10] The delivered dose to the mid-lobe of the lung was also measured with the ionization chamber (0.3 cc). In order to reduce the lung dose, a proper Cerrobend shield (1.14 cm thickness) was built.

### Lung shield

**Figure 2:**
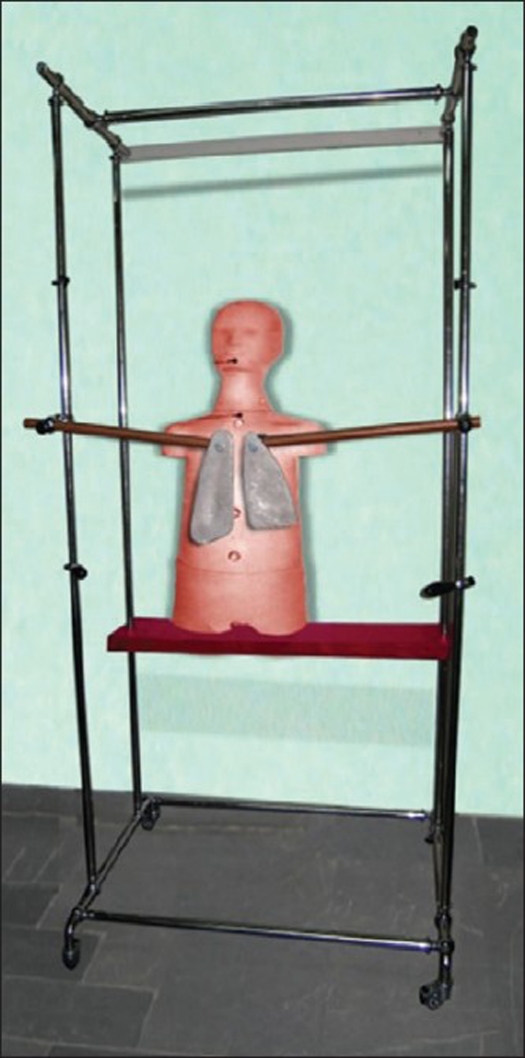
TBI stand with the lung shields

A mobile X-ray set placed at the position of ^60^Co was used to take AP and PA films covering the thoracic region of the human phantom based on TBI experimental conditions described. The films used to design the lung shield made from Cerrobend. To simplify planning and treatment, the AP and PA films were overlaid by the physicist and a combined outline was used to define the outline shield.[14,15] Additionally, in order to maintain the lung shield, a TBI stand consisting of 11 steel pieces with dimensions of 70 × 100 × 210 cm^3^ was built, which appears to be also useful for patient support [Figure 2].

## Results

The central axis depth dose data for ^60^Co for different field sizes (5 × 5 to 35 × 35 cm) at TBI treatment distance (250 cm SSD) are shown in Table 1. The results of depth dose data measured at 100 cm SSD and transformed using the Mayneord formula to the TBI distance are also shown in Table 1. The data transformed using the Mayneord formula is about 8% lower than the measured data for most of the distance at 2 to 25 cm depth. The mean differences between the calculated and measured percentage depth doses were 3.09% with a standard deviation of 2.51%, whereas in the large field used in treatments of TBI (35 × 35), this difference was 0.71% with a standard deviation of 0.96%.

**Table 1:** Measured and calculated percentage depth dose (using the Mayneord formula) at total body irradiation distance (SSD = 250 cm)

*Field size Depth (cm)*	*5 × 5*	*10 × 10*	*15 × 15*	*20 × 20*	*25 × 25*	*30 × 30*	*35 × 35*
2.5 mea	94.92	96.48	96.59	97	98.15	98.80	98.90
cal	92.82	94.27	94.89	95.30	95.51	95.72	95.82
4 mea	89.55	91.92	92.24	92.50	93.65	94.14	94.29
cal	85.38	88.55	89.82	90.56	90.98	91.30	91.51
5 mea	85.70	88.41	88.90	89.20	89.23	89.71	89.84
cal	80.90	84.78	86.39	87.47	87.90	88.33	88.65
6.5 mea	80.05	83.57	84.01	84.22	84.27	84.81	84.88
cal	73.97	78.81	81.12	82.28	83.00	83.66	84.04
7.5 mea	76.19	80.17	80.61	80.95	81.96	82.51	82.59
cal	69.65	74.91	77.48	78.93	79.83	80.50	80.95
8 mea	74.26	78.38	78.92	79.14	79.24	79.61	79.78
cal	67.42	72.85	75.55	77.24	78.25	78.93	79.38
9 mea	70.53	75.17	75.79	76.05	76.13	76.63	76.81
cal	64.43	69.15	72.00	73.83	74.98	75.78	76.35
10 mea	67.43	72.01	72.71	72.97	72.99	73.47	73.52
cal	59.40	65.43	68.68	70.54	71.81	72.62	73.32
11 mea	63.39	68.38	69.09	69.42	69.51	70.85	70.95
cal	55.78	61.79	65.08	67.32	68.61	69.56	70.38
12 mea	60.41	65.64	66.28	66.65	66.67	67.09	68.10
cal	52.25	58.33	61.91	64.06	65.61	66.56	67.40
13 mea	56.62	62.00	62.81	63.22	63.27	64.00	64.70
cal	48.91	55.17	58.8	61.10	62.67	63.88	64.61
14 mea	54.14	59.84	60.56	60.87	60.95	61.35	62.01
cal	45.78	52.04	55.72	58.18	59.77	61.12	61.98
15 mea	50.81	56.61	57.38	57.77	58.20	59.09	59.35
cal	42.93	49.02	52.88	55.37	57.11	58.36	59.23
18 mea	43.21	49.18	50.03	50.42	50.49	50.86	51.64
cal	35.32	41.01	44.90	47.48	49.30	50.72	51.63
20 mea	38.47	40.89	45.36	45.74	46.03	46.35	47.16
cal	30.81	36.39	40.11	42.77	44.49	46.09	47.15
22 mea	34.15	39.67	40.69	41.03	41.26	41.80	42.72
cal	26.99	32.14	35.94	38.52	40.42	41.77	42.72
25 mea	28.50	33.62	34.70	35.17	35.45	36.49	37.41
cal	22.23	27.03	30.65	33.03	34.86	36.35	37.40

**Figure 3:**
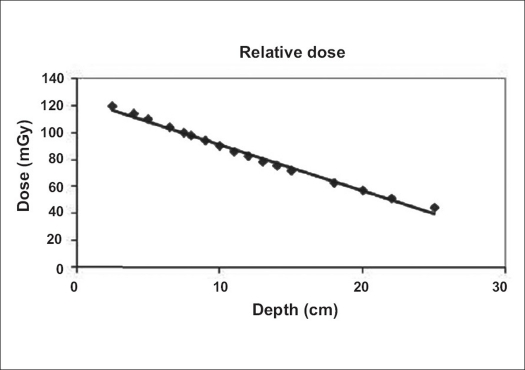
Depth dose fall-off

The depth dose fall-off from 2.5 to 25 cm depth for a 35 × 35 cm field size is shown in Figure 3.

**Figure 4:**
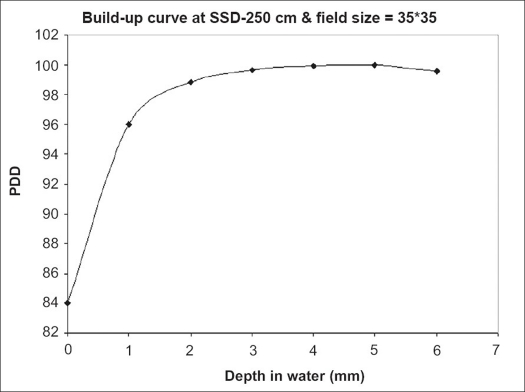
Build-up dose based on the TBI experimental conditions

The surface dose and the central axis depth dose in the build-up region for TBI treatment distance are shown in Figure 4. The results show a plateau at 5 mm and a relative surface dose of 84%.

The measured dose at the umbilicus based on SC and SP (Eq. 2) was 2.31 Gy. From this measurement, the accuracy in dose delivery at the prescribed point (umbilicus) was found to be 2.59%. Results presented in Table 2 show the effect of the doses measured at 0.5, 5 and 10 cm as a result of increasing scattering volume added in the phantom (patient) length direction. It shows that the increased scattering volume reaches a plateau at a depth of around 51 cm.

**Table 2:** The effect of adding scattering materials on the absorbed dose

*Thickness of scattering material*	*Absorbed dose (mGy) At depth of 0.5 cm*	*Absorbed dose (mGy) At depth of 5 cm*	*Absorbed dose (mGy) At depth of 10 cm*
0	119.928	105.002	86.318
3	120.063	105.378	86.759
6	120.167	105.602	87.102
9	120.302	105.853	87.291
12	120.404	105.952	87.354
15	120.507	106.078	87.543
18	120.507	106.078	87.543
21	120.507	106.239	87.543
24	120.507	106.239	87.543
27	120.507	106.239	87.543

The ratios of the measured to the calculated midplane doses based on the three algorithms are summarized in Table 3. The accuracy in the arithmetical mean algorithm was above 10%. The accuracy in the geometrical mean algorithm was within 4% and it was within 3.5% in our proposed algorithm. The mean difference between the calculated and measured doses in the first algorithm was 0.94 with a standard deviation of 0.61%; in the second algorithm, it was 2.82 with a standard deviation of 1.76% and in the third algorithm, it was 0.82 with a standard deviation of 0.54%.

**Table 3:** The ratios of measured to the calculated midplane doses based on the three algorithms

*Calculation method*	$\frac{{\text{D}}_{\text{mea}}}{{\text{D}}_{\text{cal}}}\pm \text{SD}$
Arithmetical mean algorithm	1.038 ± 0.002
Geomentrical mean algorithm	1.125 ± 0.003
Proposed algorithm	1.033 ± 0.002

Point dose measurements at anterior and posterior of human phantom are summarized in Table 4. These results show that dose uniformity at the anterior surface was within 6% and it was within 5% at the posterior surface. The delivered dose to the mid-lobe of the lung with and without the shield is presented in Table 5. It has been seen that the delivered lung dose without the shield was 14.14 Gy but by applying a proper shield (1.14 cm thickness), it has been reduced to 8.16 Gy.

**Table 4:** Point dose measurements at anterior and posterior surfaces of the human phantom

*Site*	*Anterior dose (cGy)*	*Posterior dose (cGy)*
Umbilicus	235.340	239.104
Hip	248.238	237.813
Epigastria	243.916	234.170
Supra sternal notch	236.996	243.037
Lip	230.127	250.466
Neck	236.900	238.040

**Table 5:** Delivered lung dose with and without the shield

*Lung dose*	*AP*	*PA*	*AP + PA 1 fraction*	*AP + PA 6 fractions*
Without shield (cGy)	105.800	129.850	235.650	1414
with shield (cGy)	60.990	75.070	136.060	816

## Discussion

The results in Table 1 illustrate a poor coincidence between the calculated and measured percent depth doses for small and intermediate fields. But as the collimator opening was comparable with the mean range of scattered photons, the scatter contribution to the dose reached its maximum. Hence, the calculated percent depth dose using the Mayneord formula which does not account for a change in the scatter component showed increasing agreement with the measured data. However, for the largest field, deviation is minimum and there is no significant difference between them. It can be suggested that, for the simplicity, the calculated percentage depth dose be used instead of the measured percentage depth dose. The result in Figure 3 demonstrates that the depth dose fall-off from 2.5 to 25 cm is nearly linear. Hence, a combined parallel opposed irradiation produces dose uniformity across a homogeneous transverse section of the patient.

The results of the build-up dose are shown in Figure 4. Skin sparing is undesirable for some treatment situations. For example, an adequate skin dose must be maintained for patients who have a generalized disease such as leukemia, where leukemia blast cells can be presumed to be circulating in the capillary bed immediately beneath the skin surface. Another example is neuroblastoma. This tumor is distributed widely throughout the body and in superficial bones lying less than 1 cm beneath the surface.[2] Therefore, the skin dose should be high in neuroblastomas as well. As shown in Figure 4, the skin dose is 84%, which is one of the advantages of using ^60^Co to an accelerator option in the TBI treatment, as the skin dose may be smaller with high energy accelerators. As mentioned earlier, the results based on the measurements of basic dosimetric parameters confirmed that the measured dose to the umbilicus was within 3% of the prescribed dose to the umbilicus. The accuracy and variation of the dose delivered to the umbilicus is considered to be clinically acceptable.[1] The implication of the results in Table 2 is that adding scatter material around the phantom does not changes depth doses significantly and The effect of phantom (patient) length on absorbed dose effect would be canceled. It can be seen from Table 3 that using both calculation methods, proposed and arithmetical mean algorithms, the dose agrees with the measured dose within 4%. But the geometric mean algorithm has a larger error and is not acceptable. Among the three algorithms discussed earlier, the proposed algorithm appears to be more appropriate for the determination of the midplane dose from entrance and exit measurements in TBI treatment. The results in Table 4 show that the difference between doses in the whole body relative to the umbilicus is within ±7%, which is in agreement with the results obtained by Harden.[15] The delivered dose in some areas such as the neck is higher because of the combination of reduced beam intensity and a relative lack of scattered material in this area. The lung region shows an increased dose relative to the prescribed dose to the umbilicus due to its density. However, by applying a shield, it has been reduced to 8.16 Gy which is below the tolerance level.[14] The TBI stand made in this project has the capability of being extended for shields for organs other than the lungs, for example, the eyes of patients undergoing TBI treatment.

## Conclusion

Basic dosimetric parameters which are necessary in TBI treatment were measured in this study. The proposed algorithm by this work appears to be useful with an accuracy within 2.5%. Accuracy in dose delivery was within 3% and dose uniformity through the whole body was within ±7%.

## References

[1] Khan FM (2003). The physics of radiation therapy.

[2] Purdy JR (1990). Advanced in radiation oncology physics dosimetry, treatment planning and brachytherapy. AAPM.

[3] Ribas M, Jornet N, Eudaldo T, Carabante D, Duch MA, Ginjaume M (1998). Midplane dose determination during total body irradiation using invivo dosimetry. Radiother Oncol.

[4] Van Dyk J, Galvin JM, Glasgow GP, Podgorsak EB (1986). The physical aspects of total and half body photon irradiation. AAPM.

[5] Curran WJ, Galvin JM (1989). A simple dose calculation method for total body photon irradiation. Radiat Oncol Biol Phys.

[6] Greig JR, Miller RW, Okunieff P (1996). An approach to dose measurement for total body irradiation. Radiat Oncol Biol Phys.

[7] Vrtar M (2001). Total body irradiation dosimetry of a low dose rate ^60^Co gamma field. FIZIKA B (Zagreb).

[8] Jornet M, Ribas M, Eudaldo T (1996). Calibration of semiconductor detectors for dose assessment in total body irradiation. Radiother Oncol.

[9] Hui SK, Das RK, Thomadsen B, Henderson D (2004). CT-based analysis of dose homogeneity in total body irradiation using lateral beam. J Appl Clin Med Phys.

[10] Vollans SE, Perrin B, Wilkinson JM, Rao Gattamaneni H, Deakin DP (2000). Investigation of dose homogeneity in paediatric anthropomorphic phantoms for a simple total body irradiation technique. Br J Radiol.

[11] Niroomand-Rad A (1991). Physical aspects of total body irradiation of bone marrow transplant patients using 18 MV x-rays. Int J Radiat Oncol Biol Phys.

[12] Lam WC, Lindskoug BA, order SE, Grant DG (1979). The dosimetry of ^60^Co total body irradiation. Int J Radiat Oncol Biol Phys.

[13] Hasanzadeh H, Sharafi A, Allah Verdi M, Nikoofar A (2006). Assessment of absorbed dose to thyroid, parotid and ovaries in patients undergoing Gamma Knife radiosurgery. Phys Med Biol.

[14] Jones D, Rieke JW, Madsen BL, Hafermann MD (2000). An isocentrically mounted stand for total body irradiation. Br J Radiol.

[15] Harden SV, Routsis DR, Geater AR, Thomas SJ, Taylor PJ, Marcus RE (2001). Total body irradiation using a modified standing technique: A single institution 7 year experience. Br J Radiol.

